# Introducing the Ottawa Clinical Fear of Recurrence—Self‐Report; A New Assessment Tool for Clinical Fear of Cancer Recurrence

**DOI:** 10.1002/pon.70537

**Published:** 2026-07-07

**Authors:** Lauriane Giguère, Emma Kearns, Suchithra Shenthil, Brittany Mutsaers, Cheryl Harris, Allan ‘Ben’ Smith, Gerald M. Humphris, Daniel Costa, Cary S. Kogan, Sébastien Simard, Sophie Lebel, FORWARDS

**Affiliations:** ^1^ School of Psychology University of Ottawa Ottawa ON Canada; ^2^ The Ottawa Hospital Research Institute Cancer Therapeutics Program The Ottawa Hospital Ottawa ON Canada; ^3^ The Daffodil Centre The University of Sydney a Joint Venture with Cancer Council NSW Ottawa ON Canada; ^4^ School of Medicine University of St Andrews St Andrews UK; ^5^ School of Psychology The University of Sydney Sydney Australia; ^6^ Department of Health Sciences Université du Québec à Chicoutimi (UQAC) Saguenay QC Canada

**Keywords:** cancer, fear of cancer recurrence, item response theory, oncology, patient‐reported outcome measures, psycho‐oncology

## Abstract

**Background:**

The Ottawa Clinical Fear of Recurrence—Self‐Report (OCFR‐SR) was developed to enable brief assessment of clinically significant fear of cancer recurrence (FCR) according to recently updated criteria based on expert consensus: a) high levels of worry, b) high levels of preoccupation, c) hypervigilance of bodily symptoms, and d) that last for more than 3 months.

**Aim:**

The study evaluated the OCFR‐SR item pool with the goal of optimizing its brevity and psychometric properties.

**Methods:**

The OCFR‐SR's 23 candidate items were administered to mixed cancer survivors recruited from The Ottawa Hospital along with other assessment measures within the questionnaire package. Eligible participants had to have been diagnosed with any cancer, stages I‐III, and have completed primary treatment. Exploratory factor analysis, item response theory, and internal reliability were used to optimize the number of items. Internal (MacDonald's omega and Cronbach's alpha) and test‐retest reliability (intra‐class correlation) were assessed. Finally, convergent and discriminant validity were explored (Pearson correlations and *z*‐tests).

**Results:**

A total of *N* = 296 survivors completed the questionnaire package. Seven items were selected for the final version of the OCFR‐SR. All items performed similarly on psychometric assessments. Theoretical considerations were decisive in selecting the final items. The final OCFR‐SR demonstrated satisfactory convergent and discriminant validity, and internal and test‐retest reliability.

**Conclusions:**

This study introduces the final seven‐item OCFR‐SR, enabling clinicians and researchers to assess the clinical significance of FCR according to the recently updated criteria. It is the first brief self‐report tool aimed at assessing clinical FCR.

## 1 Background

Fear of cancer recurrence (FCR) is defined as the “fear or worry that cancer will come back or progress” [1]. As treatments evolve and an increasing number of patients move on to the survivorship stage, FCR has become an important aspect of the survivorship experience. Like other mental health concerns such as anxiety, FCR can be adaptive to motivate survivors to stay up to date with their follow‐up care. However, approximately 59% of cancer survivors report that FCR impacts their daily life [2]. FCR is a major source of unmet needs for survivors.

This suggests that survivors may face challenges in accessing the care they need for managing FCR. One reason for this may be a lack of consensus on how to differentiate between adaptive and clinical levels of FCR [3]. Historically, it has been difficult for healthcare professionals to assess the severity of FCR, potentially leading to inadequate care and capacity to address survivors needs [4]. To address this, recent guidelines on the management of FCR emphasize the use of assessment tools to evaluate the specific needs of each survivor [5, 6]. Self‐report questionnaires designed to efficiently assess the severity and impact (i.e., clinical significance) of FCR could prove useful. However, researchers have only recently agreed on the criteria that indicate clinical levels of FCR [3]. Following a Delphi study with FCR experts, the following criteria were established: (a) high levels of worry, (b) persistent preoccupation with recurrence, (c) heightened sensitivity or hypervigilance to bodily symptoms, and (d) persistence of these symptoms for at least 3 months. Functional impairment, while deemed clinically useful, was not considered as an essential criterion of clinically significant FCR. Although consensus‐based clinical criteria for FCR were established in 2020, no self‐report instrument has since been developed based on these. As a result, clinicians currently rely on measures that were developed using earlier conceptualizations of FCR, which do not include all the consensus‐based clinical criteria.

For example, the nine‐item Fear of Cancer Recurrence Inventory—Short Form (FCRI‐SF) [7], the most used assessment measure, utilizes items based on severity of cancer‐related worry symptoms to assess for FCR. Although worry symptoms are important, other aspects of this construct as defined by the clinical definition may be left out (i.e., hypervigilance, duration of symptoms) [8]. Similar concerns arise for measures such as the Cancer Worry Scale (CWS) [9] or the Fear of Progression Questionnaire (FoP‐Q) [10]. The FCR4 and FCR7 have also been developed to assess the emotional, cognitive and behavioral aspects of FCR [11]. Specifically, the FCR4/7 lack an assessment of duration and persistence of symptoms. Furthermore, while it assesses self‐checking, it does so in terms of coping strategies rather than hypervigilance. These instruments prioritized brevity over comprehensive assessment of clinical criteria and conceptualized the construct as unidimensional [11]. Hypervigilance as a core feature has been largely overlooked in the above existing self‐reports [12]. Overall, these tools do not reflect the current conceptualization of clinical FCR. Moreover, most of these self‐reports’ cut‐off scores were chosen using another self‐report rather than an established semi‐structured clinical interview as a gold standard. This questionnaire‐against‐questionnaire methodology within the field may be leading to circularity and criterion contamination [13, 14]. Only the FCRI‐SF used a semi‐structure interview that the authors designed specifically for this purpose before the establishment of clinical criteria for FCR [15]. Additionally, some psychometric weaknesses have been identified for existing instruments (e.g., no test‐retest for the FCR4‐FCR7, lack of agreement on the appropriate cut‐off for the FCRI‐SF; for a detailed review of the psychometric properties of exiting FCR measures, see [16]).

To facilitate integration of self‐report measures into daily practice and maximize their utility in guiding appropriate care to survivors, there has been a push for shorter psychometrically robust tools. Recently, Podina and colleagues [17] showed that the longer the tool, the larger the association between FCR and other mental health concerns such as anxiety or depression, suggesting potential measurement artifacts. Overall, measurement significantly impacts research and clinical practice through misclassification risks leading to potential over‐ or undertreatment. Therefore, better measurement is crucial. Rather than refining existing severity scales, this study aims to assess the clinical features of FCR within a multidimensional framework that aligns theoretical models, expert‐derived criteria, and psychometric evaluation within a clinically meaningful structure, supporting identification of all aspects of clinical FCR rather than elevated fear alone.

In a previous phase of this study, an initial pool of 23 items for the Ottawa Clinical Fear of Recurrence—Self‐Report (OCFR‐SR) was developed based on content validity using pilot testing with the target population and assessment by content experts [18]. The current phase consists of optimizing the number of items and exploring the psychometric properties of the finalized OCFR‐SR. This study will be followed by a subsequent phase for clinical threshold identification using a newly developed clinical interview [18].

### 1.1 Objectives

This article reports the outcome of optimizing the number of items in the OCFR‐SR from its current 23‐item pool to produce a concise and clinically useful tool that can rapidly assess clinical FCR capturing all of the construct's aspects. There were three specific study objectives:

1. Conduct an exploratory factor analysis (EFA), item response theory (IRT) analysis and internal consistency analysis, to optimize the number of items on the instrument;
2. Report the finalized OCFR‐SR's incremental, discriminant and convergent validity, to determine how well it is capturing the underlying construct of FCR;
3. Explore the finalized OCFR‐SR's test‐retest and internal reliability.

Based on previous research, we hypothesized that the final OCFR‐SR would; a) fit a 4‐factor model as per clinical FCR criteria (preoccupation, worry, hypervigilance, and impairment) with at least 2‐4 items per factor [3, 19]; b) demonstrate convergent validity through strong positive correlations (0.70 < *r* ≤ 1.00) between OCFR‐SR scores and existing assessment tools for FCR, hypervigilance, and anxiety, as well as established correlates of high FCR, namely younger age, female sex, lung and breast cancer types [2, 20]; c) demonstrate discriminant validity through a small to moderate or negative correlation with general life satisfaction (−0.60 < *r* ≤ 0.20) and depressive symptoms (0.00 < *r* ≤ 0.60) [2, 20] and non‐significant correlations with lower education, treatment type, ethnicity, income, marital status, treatment end‐date, cancer stage and time since diagnosis [2, 20]; and d) have high internal consistency (≥ 0.8) [21] and test‐retest reliability (intra‐class correlation > 0.75) [22].

## 2 Methods

The study was approved by the Ethics Board of The Ottawa Hospital (#20210304‐01H) and performed in accordance with the Declaration of Helsinki's ethical standards.

### 2.1 Participants

Lists of cancer survivors who had completed treatment at The Ottawa Hospital were screened for potentially eligible participants. Those who were 18 or older, appeared to have completed their primary treatment, were diagnosed with any cancer (stages I‐III), and had provided consent to be contacted for research purposes were sent a recruitment letter detailing the study and containing a QR code and link to the questionnaire package on Qualtrics. The minimum sample size required was at 230 participants, at least 10 times the number of participants per items on the questionnaire being assessed for EFA and IRT (i.e., OCFR‐SR's 23 potential items) [23]. However, to account for missing data, we aimed for 300 participants.

A sub‐sample of 50 volunteers among those completing the questionnaire package participated in test‐retest reliability by completing the questionnaire package again two weeks later.

### 2.2 Measures

The questionnaire package contained a consent form, a demographic and medical characteristics section and seven measures of FCR and related concepts [24]. This questionnaire package took approximately 20 min to complete.

#### 2.2.1 Ottawa Clinical Fear of Recurrence—Self‐Report (OCFR‐SR) [18]

This instrument has a pool of 23 potential items and targets each criterion of clinical FCR by dividing items into sections regarding worry, preoccupation, hypervigilance and impairment. Persistence is assessed using a Likert scale of frequency.

#### 2.2.2 Convergent Validity

To assess convergent validity, or the extent to which FCR correlates with other related constructs based on theory or previous research [25], demographic/clinical characteristics and the following instruments were included in the questionnaire package.

*Fear of Cancer Recurrence Inventory Short Form (FCRI‐SF)* [7] is a 9‐item short‐form of the 42‐item Fear of Cancer Recurrence Inventory. This short form is also the severity subscale of the scale. Higher scores indicate higher levels of FCR and can range from 0 to 36.

*Cancer Worry Scale (CWS)* [26] is a 6‐item assessment of cancer‐related worries and their impact on everyday life. Higher scores indicate more severe worries. Scores can range from 8 to 32.

*PROMIS‐Anxiety‐8a (PROMIS‐A‐8a)* [27, 28, 29] is an 8‐item self‐report of anxiety in adults and those with chronic illnesses. It assesses fear, anxious misery, hyperarousal, and somatic symptoms related to arousal over the previous 7 days. Score can range from 8 to 40.

*Bodily Threat Monitoring Scale (BTMS)* [30] is a 19‐item self‐report assessing hypervigilance and threatening interpretations of bodily sensations. Scores can range from 0 to 76.

#### 2.2.3 Discriminant Validity

For discriminant validity, or the extent to which the new scale does not correlate with instruments assessing distinct constructs, demographic characteristics and the following instruments were included in the questionnaire package.

*PROMIS‐Depression‐8a (PROMIS‐D‐8a)* [28, 29, 31] is an 8‐item self‐report measuring depression in adults and those with chronic illnesses by assessing negative mood, views of self, social cognition, and decreased positive affect and engagement over the previous 7 days. Scores can range from 8 to 40.

*PROMIS General Life Satisfaction‐SF5a (PROMIS‐GLS)* [32] is a 5‐item assessment of life satisfaction in adults. The measure assesses whether the individual's life has been satisfactory according to their values and expectations. Scores can range from 5 to 33.

#### 2.2.4 Procedure

Potential participants receiving the recruitment letter by mail could complete the questionnaire online or contact the research team for a paper copy. Data were screened weekly to ensure proper collection, track completeness of responses and ensure eligibility of participants completing the questionnaire. Recruitment letters were issued until approximately 300 eligible participants had responded.

### 2.3 Statistical Methods

#### 2.3.1 Exploratory Factor Analysis

The Kaiser method and Bartlett's test of sphericity were used to test for the adequacy of using factor analysis based on the sample. The closer to 1.00 Kaiser‐Meyer‐Olkin's was, the more adequate the use of a factor analysis for this sample [33, 34]. In addition, Bartlett's test of sphericity was hypothesized to be significant to demonstrate independence. The exploratory factor analysis was conducted using the maximum likelihood method of extraction with the Oblimin oblique rotation as the latent constructs describing the items were expected to be conceptually related. Factors were extracted based on the conventional rule of eigenvalues > 1. Items loadings of < 0.30 on their respective factor or double loading on two factors were removed from the item pool.

#### 2.3.2 Item Response Theory

Item response theory (IRT) is a psychometric theory founded on the probability of an item being answered correctly as a function of the non‐observable trait or ability being assessed (latent variable) [35]. A polytomous model was used since each item contains more than two response options [36]. Constrained (i.e., constant discrimination parameter across all items; Rasch modeling or 1PL) [37] and non‐constrained models (i.e., varying discrimination parameter across all items; 2PL or 3PL models) [37] were generated using the ‘ltm’ package in *R* and then tested for best fit and compared using an ANOVA. Item response curves (or item characteristic curves), difficulty parameters, and discrimination parameters were used to illustrate the results and interpret their implications. Difficulty parameters indicate the level of latent trait needed to reach a 50% probability of endorsing a response option [36]. On a Likert scale (in this case 1–5), each response option should require a gradually higher level of latent trait. Relatedly, discrimination parameters represent the ability of an item to differentiate between those who highly endorse the latent trait and those who do not. Previous studies have used *a* > 1.7 discrimination parameter as an indication of satisfactory discrimination [11, 38]. Item characteristic curves plot these two parameters.

A consecutive unidimensional item response theory (UIRT) method was chosen instead of a multidimensional IRT (MIRT) approach. Existing literature indicates that a consecutive UIRT method yields accuracy comparable to that of a MIRT [39, 40].

#### 2.3.3 Reliability

Intra‐class correlation computed by a single‐rating, absolute‐agreement, two‐way mixed effect was used for test‐retest. For internal reliability, both Cronbach's alpha and MacDonald's omega were calculated. Cronbach's alpha was used for the measure as a whole and its subscales. It was also used to assess for the scale's reliability if an item was deleted. Considering Cronbach's alpha limitations with multidimensional scales, MacDonald's omega was prioritized for the reliability of the scale as a whole [41]. It is based on factor analysis and offers two coefficients: omega hierarchical and omega total. Omega hierarchical represents the reliability of the general factor causing variance. It controls for the variance caused by specific factors. Omega total represents the reliability of the underlying construct of the subscale, considering all factors in the model. It tends to be higher than Cronbach's. A value of ≥ 0.8 for both alpha and omega will be considered necessary for good internal consistency [42, 43].

#### 2.3.4 Validity

Pearson correlations were used to assess for convergent and discriminant validity. Given the frequent occurrence of statistically significant positive correlations among measures of FCR, anxiety, and depressive symptoms, differences in the strength of these correlations were examined using chi‐square and z‐tests [44]. To control for multiple comparisons, a significance threshold of 0.01 was applied to determine the statistical significance of the Pearson correlations. Alpha was set to 0.05 for all other tests.

## 3 Results

### 3.1 Participants

Data was collected from October 2022 to February 2024. A total of 1588 recruitment letters were sent to potentially eligible cancer survivors having completed treatments at The Ottawa Hospital. A total of 676 potential participants were invited to take part in this study while 912 were not (i.e., could not reach/wrong address (*n* = 790), ineligible (*n* = 86), or deceased (*n* = 36)). A total of 296 (43.7%) eligible participants provided consent and submitted a complete response (i.e., provided at least demographic and medical characteristics as well as answers to the OCFR‐SR). Table 1 presents the sample's demographic and medical characteristics.

**TABLE 1:** Demographic and medical characteristics of sample (*N* = 296).

Demographic characteristic		Total *(%)*
Marital status	Married/Common law	226 (76.4)
	Divorced separated	31 (10.5)
	Widowed	21 (7.1)
	Single, never married	18 (6.1)
Education	Graduate school	53 (17.9)
	University/college	142 (48.0)
	Some university/college	52 (17.6)
	High school	40 (13.5)
	Some high school	8 (2.7)
	Elementary school	1 (0.3)
Occupational status	Retired	169 (56.9)
	Employed full‐time	78 (26.3)
	Employed part‐time by choice	21 (7.1)
	Unemployed due to illness	14 (4.8)
	Employed part‐time due to illness	5 (1.7)
	Homemaker	4 (1.3)
	Student	2 (0.7)
	Unemployed	1 (0.3)
Ethnic background	White	273 (92.2)
	Other	11 (3.7)
	Asian	5 (1.7)
	Indigenous/Metis	4 (1.4)
	African‐canadian	2 (0.7)
	Hispanic	2 (0.7)
Annual income	Greater than $100,000	62 (20.9)
	$81,000‐$100,000	54 (18.2)
	$61,000‐$80,000	58 (19.6)
	$41,000‐$60,000	58 (19.6)
	$21,000‐$40,000	43 (14.5)
	$0,000‐$20,000	21 (7.1)
Age	[21–95]	64.2 (12.4)
Sex	Female	159 (53.7)
	Male	136 (45.9)
	Prefer not to say	1 (0.3)
Cancer type	Breast	82 (27.7)
	Prostate/testicular	42 (14.2)
	Colorectal/bowel	34 (11.5)
	Lymphoma	23 (7.8)
	Melanoma	18 (6.1)
	Other	17 (5.7)
	Kidney/bladder	16 (4.4)
	Lung	13 (3.7)
	Head/neck	11 (3.7)
	Leukemia	9 (3.0)
	Hepatobiliary disease (pancreas, liver, bile duct)	9 (3.0)
	Endometrial	7 (2.4)
	Cervical	6 (2.0)
	Ovarian	4 (1.4)
	Brain	4 (1.4)
	Esophageal	3 (1.0)
	Multiple myeloma	3 (1.0)
	Stomach	1 (0.3)
Time since diagnosis (Years)	[0–35]	5.8 (5.4)
Time since end of treatment (Years)	[0–35]	4.0 (4.1)
Treatment type	Had surgery	220 (74.3)
	Had radiation	155 (52.4)
	Had chemotherapy	120 (40.5)
	Had adjuvant therapy	60 (20.3)
	Had other treatment	39 (13.2)

### 3.2 Item Selection

#### 3.2.1 EFA

Bartlett's test (*x* = 8718L.78, *p* < 0.001) and Kaiser‐Meyer‐Olkin's measure of sampling adequacy (*x* = 0.964) indicated that this sample was appropriate for EFA. Communalities ranged from 0.58 to 0.91. Three factors were extracted. The first factor (preoccupation/worry) explained 68.83% of the variance. The other two factors (hypervigilance and impairment factors) explained 4.82% and 7.5% of the variance respectively. Together, these factors explained 81.14% of the total variance. Table 2 presents the pattern matrix and commonalities.

**TABLE 2:** Pattern matrix and commonalities.

Item	Factor 1	Factor 2	Factor 3	Commonalities
1. I have been distressed when thinking about the possibility of having a cancer recurrence	0.873			0.85
2. I have images (mental pictures) of myself being ill due to cancer coming back	0.758			0.77
3. I have concerns about the cancer coming back	0.926			0.86
4. I have trouble thinking about other things in my life because of concerns about my cancer recurring	0.504	0.505		0.88
5. It is difficult to take my mind off thoughts or concerns related to the possibility of having a cancer recurrence	0.576	0.459		0.87
6. I worry about the possibility of cancer recurrence	0.999			0.91
7. I worry about the possibility of my cancer coming back	0.936			0.91
8. I am afraid of a cancer recurrence	0.863			0.86
9. I am anxious about the possibility of cancer recurrence	0.803			0.85
10. My worries about cancer recurrence overwhelm me	0.312	0.547		0.85
11. I find it difficult to control my worries about cancer recurrence	0.418	0.598		0.89
12. When I notice an unpleasant feeling in my body (e.g., pain, ache, or other uncomfortable sensation) I find it difficult to think of anything else	0.389		0.362	0.79
13. I spend a lot of time examining myself to see if I have any physical signs of cancer			0.915	0.83
14. I check my body for signs that my cancer has come back			0.945	0.79
15. I spend a lot of time seeking out information about signs of cancer recurrence			0.559	0.58
16. When I notice an ache, pain, or other unpleasant sensations in my body, I think it is a sign that my cancer has come back	0.339		0.482	0.77
17. I worry about my cancer coming back in between my appointments	0.458		0.392	0.74
18. I am unable to work because of my concerns about cancer coming back		0.836		0.66
19. I have difficulty concentrating on what I am doing because of concerns about cancer recurrence		0.900		0.84
20. My thoughts or fears about the possibility of cancer recurrence disrupt my work or everyday activities		0.914		0.83
21. I have trouble sleeping because I cannot get thoughts about cancer out of my mind		0.590		0.75
22. Fear of cancer recurrence interferes with my daily life (e.g., at work, my relationships)		0.800		0.84
23. My worries about cancer coming back disrupt my relationships with family, friends, and others who are close to me		0.691		0.71
Eigenvalues	15.83	1.72	1.11	
% of variance	68.83%	7.50%	4.82%	

Items 1‐3 and 6‐9 were chosen as potential representatives of the first factor (preoccupation/worry). These items' loadings were > 0.3 and loaded on a single factor. Specifically, items 3, 6, and 7 had the highest loadings (eigenvalue > 0.900). Items 13–15 were potential representatives of the hypervigilance factor. Finally, items 18–23 were chosen for the impairment factor based on the same reasoning. Items 13, 14, 19, and 20 all had eigenvalues > 0.900.

#### 3.2.2 IRT

The worry/preoccupation factor's structure fitted a 2PL model. The non‐constrained model showed significant improvement over the constrained model (F(6) = 38.34, *p* < 0.001). All items showed satisfactory and similar discrimination (*a* > 1.7 on all items) and difficulty thresholds. ICCs also appeared to confirm this similarity. For the hypervigilance to bodily symptoms factor, a Rasch model fit the data best (F(2) = −871.26, *p* = 1). Difficulty parameters varied. Specifically, item 15 appeared to necessitate a higher level of the latent trait than items 13 and 14. ICCs also appeared to confirm this; therefore item 15 was discarded. For the impairment factor, a Rasch model was fit to the data. The non‐constrained model did not show a significant improvement to the constrained model (F(5) = −60.35, *p* = 1) The constant discrimination parameter remained satisfactory (*a* > 1.7). However, all items had similar and satisfactory difficulty thresholds which made it more difficult to guide item selection.

The resulting discrimination estimates and difficulty thresholds for each item are shown in the table below (Table 3). Item characteristic curves can be found in Supporting Information S1: Appendix A.

**TABLE 3:** IRT discrimination and difficulty parameters.

Item	Factor	Discrimination parameter (α)	*d*1	*d*2	*d*3	*d*4
1. I have been distressed when thinking about the possibility of having a cancer recurrence	1	3.474	−0.791	0.481	1.198	2.003
2. I have images (mental pictures) of myself being ill due to cancer coming back	1	3.456	−0.364	0.671	1.233	2.043
3. I have concerns about the cancer coming back	1	2.287	−0.899	0.205	1.127	2.103
6. I worry about the possibility of cancer recurrence	1	3.306	−0.818	0.387	1.120	2.118
7. I worry about the possibility of my cancer coming back	1	3.542	−0.715	0.338	1.142	2.092
8. I am afraid of a cancer recurrence	1	3.552	−0.645	0.356	1.149	1.989
9. I am anxious about the possibility of cancer recurrence	1	3.777	−0.496	0.515	1.112	1.876
13. I spend a lot of time examining myself to see if I have any physical signs of cancer	3	3.655	−0.430	0.495	1.310	1.923
14. I check my body for signs that my cancer has come back	3	3.655	−0.659	0.309	1.139	1.892
15. I spend a lot of time seeking out information about signs of cancer recurrence	3	3.655	−0.248	0.845	1.509	2.201
18. I am unable to work because of my concerns about cancer coming back	2	3.489	0.914	1.632	1.938	2.336
19. I have difficulty concentrating on what I am doing because of concerns about cancer recurrence	2	3.489	0.392	1.315	1.706	2.486
20. My thoughts or fears about the possibility of cancer recurrence disrupt my work or everyday activities	2	3.489	0.454	1.318	1.865	2.463
21. I have trouble sleeping because I cannot get thoughts about cancer out of my mind	2	3.489	0.080	1.178	1.867	2.495
22. Fear of cancer recurrence interferes with my daily life (e.g., at work, my relationships)	2	3.489	0.386	1.175	1.754	2.637
23. My worries about cancer coming back disrupt my relationships with family, friends, and others who are close to me	2	3.489	0.474	1.265	1.954	2.679

#### 3.2.3 Reliability

Cronbach's alpha for the scale if an item is deleted is represented in Table 4. The largest impact can be seen when removing items 13 or 14, meaning that these are important to the internal reliability of the scale.

**TABLE 4:** Impact of each item on the scale's internal consistency.

Items	Cronbach's alpha if item deleted
1. I have been distressed when thinking about the possibility of having a cancer recurrence	0.97
2. I have images (mental pictures) of myself being ill due to cancer coming back	0.98
3. I have concerns about the cancer coming back	0.97
6. I worry about the possibility of cancer recurrence	0.97
7. I worry about the possibility of my cancer coming back	0.97
8. I am afraid of a cancer recurrence	0.97
9. I am anxious about the possibility of cancer recurrence	0.97
13. I spend a lot of time examining myself to see if I have any physical signs of cancer	0.78
14. I check my body for signs that my cancer has come back	0.81
15. I spend a lot of time seeking out information about signs of cancer recurrence	0.93
18. I am unable to work because of my concerns about cancer coming back	0.94
19. I have difficulty concentrating on what I am doing because of concerns about cancer recurrence	0.93
20. My thoughts or fears about the possibility of cancer recurrence disrupt my work or everyday activities	0.93
21. I have trouble sleeping because I cannot get thoughts about cancer out of my mind	0.94
22. Fear of cancer recurrence interferes with my daily life (e.g., at work, my relationships)	0.92
23. My worries about cancer coming back disrupt my relationships with family, friends, and others who are close to me	0.94

### 3.3 Additional Theoretical Considerations

For the preoccupation/worry factor, items 6, 7, 8, and 9 were all similar in their content and psychometric properties. The research team decided to retain only one. Item 6 was deemed to be the most representative of FCR by the research team. Item 1 was deemed essential to assessing clinical FCR by the research team. In addition, item 2 represented more specific intrusive thoughts, which is also relevant to screening for clinical FCR. For example, a similar item has been used in the FCRI [7]. Item 3 focused on concerns about recurrence which may be difficult for survivors to distinguish from item 6, 7, 8, or 9. Items 18, 21, and 23 may be too specific and may not apply to all survivors (e.g., particular to employed individuals). Item 20 was too specific. Item 22 was deemed more general and inclusive of difficulties of different areas of functioning (i.e., work, activities of daily living, relationships).

### 3.4 Finalized Scale

#### 3.4.1 Items Selected

Based on the above results and theoretical considerations, the research team selected seven items to constitute the final version of the OCFR‐SR. Items 1, 2, and 6 represent the worry/preoccupation factor, items 13–14 represent the hypervigilance factor and items 19 and 22 represent the impairment factor.

#### 3.4.2 Reliability

Internal reliability was satisfactory. The scale as a whole demonstrated high reliability on Cronbach's alpha (*a* = 0.93), omega hierarchical (*w* = 0.84) and omega total (*w* = 0.97). The subscales also demonstrated high reliability with a Cronbach's alpha of *a* = 0.93 for the preoccupation/worry factor, *a* = 0.93 for the hypervigilance subscale and *a* = 0.87 for the impairment subscale.

The test‐retest interclass correlation of the self‐report within 95% CI was 0.892 [0.821–0.902], indicating good reliability [22]. On average, participants completed the self‐report 16 days following initial completion.

#### 3.4.3 Validity

##### 3.4.3.1 Convergent

The results support our hypotheses for convergent validity of the OCFR‐SR scale, with strong positive correlations between the OCFR‐SR with the FCRI‐SF (*r* = 0.83, *p* < 0.01), the CWS (*r* = 0.84, *p* < 0.01), and the PROMIS‐A‐8a (*r* = 0.80, *p* < 0.01) and the BTMS (*r* = 0.74, *p* < 0.01). The OCFR‐SR also had small to moderate negative correlations with younger age (*r* = −0.32, *p* < 0.01) and female sex (*r* = −0.16, *p* = 0.01) (see Table 5).

**TABLE 5:** Descriptive statisics and correlations for convergent and discriminant validity of the OCFR‐SR.

Variable	M (SD)	1	2	3	4	5	6	7	8	9	10	11	12	13	14	15
1. OCFR‐SR	14.5 (6.4)	—														
Convergent validity																
2. Age	64.2 (12.4)	**−0.32** **	—													
3. Gender		**−0.16** **	**0.20** **	—												
4. FCRI‐SF	23.6 (7.5)	**0.83** **	**−0.32** **	**−0.21** **	—											
5. CWS	11.6 (4.1)	**0.84** **	**−0.28** **	**−0.19** **	**0.84** **	—										
6. PROMIS‐A‐8a	15.8 (7.8)	**0.80** **	**−0.34** **	**−0.22** **	**0.74** **	**0.80** **	—									
7. BTMS	39.7 (16.7)	**0.74** **	**−0.26** **	**−0.21** **	**0.67** **	**0.67** **	**0.73** **	—								
Discriminant validity																
8. Marital status (Coded)		0.01	−0.01	0.09	0.04	−0.01	−0.06	0.12	—							
9. Highest education level (Coded)		−0.04	**−0.16** **	0.03	0.04	−0.03	−0.02	−0.02	0.04	—						
10. Ethnic background (Coded)		0.07	**−0.15** **	0.01	0.02	0.03	**0.13** *	0.09	−0.06	0.09	—					
11. Annual income level (Coded)		0.01	**−0.19** **	0.02	0.06	0.00	0.01	0.03	**0.18** **	**0.32** **	0.00	—				
12. Time since diagnosis	5.8 (5.4)	0.04	**0.19** **	−0.09	0.01	0.09	0.06	0.12	−0.08	**−0.14** *	0.01	−0.08	—			
13. Time since treatment completion	4.0 (4.1)	−0.02	0.12	**−0.18** **	0.01	0.09	0.01	0.12	−0.05	−0.04	−0.01	−0.04	**0.73** **	—		
14. Cancer stage		0.15	0.04	0.14	0.15	**0.16** *	0.07	0.13	−0.03	−0.06	−0.11	**−0.16** *	−0.07	**−0.24** **	—	
15. PROMIS‐D‐8a	13.8 (7.0)	**0.66** **	**−0.25** **	−0.05	**0.60** **	**0.67** **	**0.75** **	**0.61** **	−0.10	−0.06	**0.16** **	−0.05	0.02	−0.07	0.07	—
16. PROMIS‐GLS‐S5a	23.0 (6.1)	**−0.39** **	**0.16** *	0.08	**−0.38** **	**−0.42** **	**−0.36** **	**−0.42** **	0.14	0.02	−0.11	0.05	0.01	−0.03	−0.03	**−0.52** **

*Note:* Significant correlations are in boldface. Demographic variables were dichotomized: marital status (Single vs. In a relationship), education (Less than University vs. University or Higher), ethnicity (Caucasian vs. Non‐Caucasian), and income ($0–60,000 vs. $61,000+). Cancer stage was included as a medical characteristic; however, responses were heterogeneous and inconsistent. Of 296 participants, only 166 participants provided clear and valid responses, while others were either unaware of their cancer stage or reported stages outside the conventional 1–4 classification that were particular to their cancer type.

^*^ *p* < 0.05.

^**^ *p* < 0.001 (two‐tailed).

Furthermore, a one‐way ANOVA revealed a significant difference in FCR levels between cancer types (F(16, 266) = 2.2, *p* = 0.01). However valid comparisons were not possible due to small sample size numbers of each cancer type.

##### 3.4.3.2 Discriminant

Discriminant validity analyses showed small to moderate correlations between the OCFR‐SR and demographic characteristics (see Table 5). A moderate, negative correlation was found with PROMIS‐GLS‐S5a (*r* = −0.39, *p* < 0.01), consistent with expectations. The OCFR‐SR indicated positive, moderate correlations with the PROMIS‐D‐8a (*r* = 0.66, *p* < 0.01), suggesting a stronger‐than‐expected association with depressive symptoms (see Table 5). Other FCR instruments behaved similarly, with positive, moderate correlations with the PROMIS‐D‐8a (see Table 5). However, a z‐test indicated that these associations were significantly weaker than each FCR measure's correlation with anxiety (OCFR‐SR: *z* = 5.8, *p* < 0.01; FCRI‐SF: *z* = 5.0, *p* < 0.01; CWS: *z* = 5.1, *p* < 0.01).

## 4 Discussion

This study's objective was to optimize the OCFR‐SR and report its psychometric properties. To select items from the original 23‐item pool to its current 7, an EFA, IRT and internal consistency analysis were performed and theoretical understanding of clinical FCR was considered. In addition, the final instrument's validity and reliability were assessed. The OCFR‐SR demonstrates strong psychometric performance while directly operationalizing consensus‐based clinical criteria. The finalized OCFR‐SR can be found in Supporting Information S1: Appendix B.

The collapse of the worry and preoccupation subscales indicated that these two concepts may be too difficult to distinguish in participants' answers. This may be a result of the way the scientific community as opposed to the patient/survivor community understands FCR. Likewise, it may be a result of these concepts constituting one continuum of the same phenomenon. On the other hand, the items included in the initial item pool may have been too similarly worded to properly capture the difference between preoccupation and worry. This can be further investigated using a confirmatory factor analysis in a different sample. Furthermore, considering the collapse of worry and preoccupation into one factor and the higher eigenvalue this new factor harbored, the research team chose to represent it with more items that the other two factors.

The choice of removing item 15 was consistent with the definition of hypervigilance/hypersensitivity [3]. Specifically, hypervigilance is understood as elevated attention to symptoms or bodily sensations possibly related to cancer, triggering worries. The research team concluded that instead of hypervigilance, item 15 assessed reassurance‐ and information‐seeking. Impairment was added to this assessment tool to detect clinical FCR more easily, despite its optionality in the clinical definition [3]. Therefore, items representing this factor should be general enough and capture several areas of daily living and thus be inclusive of all forms of impairment broadly speaking.

Preliminary validation results were consistent with our hypotheses, suggesting good convergent and discriminant validity [2, 45]. While we had initially hypothesized lower correlations with depression assessments, some studies have suggested a moderate and positive association between FCR and depression, particularly for breast, colorectal and prostate cancer survivors [45]. In line with this, the OCFR‐SR was moderately associated with depressive symptoms; however, further analysis suggested that this association was significantly weaker than its correlation with anxiety, reinforcing the view of anxiety as a more central aspect of FCR [2]. Overall, the OCFR‐SR behaved similarly to the other established FCR self‐reports in our study. That is, correlations with anxiety and depressive symptoms were similar for all instruments, suggesting that there may have been sample characteristics at play. In addition, the OCFR‐SR's higher correlation with the BTMS, compared with the FCRI‐SF's or CWS's, suggests that this may have been due to the inclusion of hypervigilance‐specific items. Previous research has demonstrated the predictive value of hypervigilance and bodily threat monitoring for FCR and these results confirm its relevance to the assessment of FCR [12].

### 4.1 Study Limitations

This study has limitations. First, pertaining to IRT, multiple models and iterations of each subscale were tested. Also, items were added and removed at each step to test different versions of the scale and attempt to find the best fitting model for the scale. However, it remains possible that there may be a better fitting model that was omitted from this analysis due to the particularities of this sample. It is worth noting that all items performed well psychometrically, and that the final decision rested mostly on theoretical and practical considerations by the research team. Furthermore, although this sample had heterogeneous cancer types, age, income brackets, occupational statuses, and educational achievements, it lacks the same variability for ethnicities, race, and gender. To better assess the validity of this self‐report, a sample more representative of the diversity within Canada's population is crucial. Finally, the OCFR‐SR shares similarities with the FCR‐7 [11] which covers some of the clinical criteria. However, the differences are multifold. Both instruments serve different purpose (clinical classification vs. severity measurement), rely on different conceptual frameworks (criteria‐based vs. unidimensional), assess different aspects of FCR (hypervigilance vs. behavioral responses to worry, a feature that was not retained in the consensus‐based Delphi study) and used different validation approaches (interview‐derived cut‐off vs. distribution‐based cut‐off). However, their psychometric properties should be compared in future studies, as no study to date as directly compared the predictive validity of any FCR measure, an urgent gap in the literature.

### 4.2 Clinical Implications

The OCFR‐SR is likely to be a useful tool for assessing clinically significant FCR in practice, due to five key differences from existing FCR measures: (1) its primary purpose is to approximate clinical classification based on consensus‐defined criteria (worry, preoccupation, hypervigilance, persistence, and functional interference); (2) it was developed after and aligned with these Delphi‐derived expert criteria for clinical FCR while other tools were developed before the creation of these criteria; (3) it is embedded within a stepped‐care assessment framework, as recommended by recent FCR guideline (ADD REF); (4) it will derive its clinical thresholds from a purpose‐designed semi‐structured clinical interview serving as a gold standard; and (5) it is brief, open‐access, and designed for integration into routine oncology workflows.

As the OCFR‐SR cannot substitute a full clinical assessment, a full clinical semi‐structured is in the final stages of validation. However the OCFR‐SR can be used for: (1) initial assessments in clinical practice, to triage and determine who may need referral or more intensive support; (2) research studies evaluating interventions when the goal is to measure clinically significant FCR as an inclusion criterion or outcome; (3) longitudinal monitoring where the goal is to track changes in clinically meaningful FCR over time or in the context of stepped‐care models. Finally, the intended users of the OCFR‐SR are (1) clinicians and decision‐makers in various healthcare settings, especially those that want to identify cancer patients and survivors who present with the features of clinical FCR; (2) researchers who need a brief, psychometrically valid self‐report tool that aligns with consensus‐based clinical criteria, and 3) stepped‐care program developers who want a measure that is integrated with an assessment system including screening, self‐report and clinical interview, as the OCFR‐SR is part of a family of instruments that includes all three [18].

## 5 Conclusion

Overall, this study showed that the OCFR‐SR, in its final version, has strong psychometric properties. Dimensional measures such as the FCRI‐SF and FCR7 remain useful for research examining variability in FCR severity and its correlates, whereas the OCFR‐SR may be advantageous when the objective is to approximate the presence of clinically significant FCR according to consensus‐based criteria.

## Funding

This work was supported by internal funding from the University of Ottawa's Faculty of Social Sciences to Dr. Sophie Lebel, and funding from the Ontario Graduate Scholarship (OGS) and Fonds de recherche du Québec en santé (FRQS) awards for Lauriane Giguère.

## Conflicts of Interest

The authors declare no conflicts of interest.

## Supporting information


Supporting Information S1

## Data Availability

The data that support the findings of this study are available from the corresponding author upon reasonable request.

## References

[1] S. Lebel , G. Ozakinci , G. Humphris , et al. “From Normal Response to Clinical Problem: Definition and Clinical Features of Fear of Cancer Recurrence,” Supportive Care in Cancer 24, no. 8 (August 2016): 3265–3268, 10.1007/s00520-016-3272-5. 27169703

[2] Y. L. Luigjes‐Huizer , N. M. Tauber , G. Humphris , et al., “What Is the Prevalence of Fear of Cancer Recurrence in Cancer Survivors and Patients? A Systematic Review and Individual Participant Data Meta‐Analysis,” Psycho‐Oncology (April 2022). 10.1002/pon.5921 PMC9321869 35388525

[3] B. Mutsaers , P. Butow , A. Dinkel , et al. “Identifying the Key Characteristics of Clinical Fear of Cancer Recurrence: An International Delphi Study,” Psycho‐Oncology 29, no. 2 (2020): 430–436, 10.1002/pon.5283. 31713279

[4] B. Mutsaers , G. Jones , N. Rutkowski , et al. “When Fear of Cancer Recurrence Becomes a Clinical Issue: A Qualitative Analysis of Features Associated With Clinical Fear of Cancer Recurrence,” Supportive Care in Cancer 24, no. 10 (2016): 4207–4218, 10.1007/s00520-016-3248-5. 27169700

[5] S. Lebel , C. Zwaal , L. Craig , et al., “Fear of Cancer Recurrence Guideline. Toronto (ON): Ontario Health (Cancer Care Ontario); 2024 March 1. Program in Evidence‐Based Care Guideline No.: 19‐7, https://www.cancercareontario.ca/en/guidelines‐advice/types‐of‐cancer/75576.

[6] A. B. Smith , A. Girgis , N. Taylor , et al. “Step‐By‐Step: A Clinical Pathway for Stepped Care Management of Fear of Cancer Recurrence—Results of a Three‐Round Online Delphi Consensus Process With Australian Health Professionals and Researchers,” Journal of Cancer Survivorship 20, no. 2 (October 2024): 766–780 [Internet] [cited 2025 Feb 14], 10.1007/s11764-024-01685-1. 39375279 PMC12988909

[7] S. Simard and J. Savard , “Fear of Cancer Recurrence Inventory: Development and Initial Validation of a Multidimensional Measure of Fear of Cancer Recurrence,” Supportive Care in Cancer 17, no. 3 (April 2008): 241–251, 10.1007/s00520-008-0444-y. 18414902

[8] A. B. Smith , D. Costa , J. Galica , et al. “Spotlight on the Fear of Cancer Recurrence Inventory (FCRI),” PRBM 13 (December 2020): 1257–1268, 10.2147/prbm.s231577. PMC7762428 33376421

[9] J. A. E. Custers , L. Kwakkenbos , M. van de Wal , J. B. Prins , and B. Thewes , “Re‐validation and Screening Capacity of the 6‐Item Version of the Cancer Worry Scale,” Psycho‐Oncology 27, no. 11 (2018): 2609–2615, 10.1002/pon.4782. 29843189

[10] P. Herschbach , P. Berg , A. Dankert , et al., “Fear of Progression in Chronic Diseases: Psychometric Properties of the Fear of Progression Questionnaire,” Journal of Psychosomatic Research 58, no. 6 (June 2005): 505–511, 10.1016/j.jpsychores.2005.02.007. 16125517

[11] G. M. Humphris , E. Watson , M. Sharpe , and G. Ozakinci , “Unidimensional Scales for Fears of Cancer Recurrence and Their Psychometric Properties: The FCR4 and FCR7,” Health and Quality of Life Outcomes 16, no. 1 (February 2018): 30, 10.1186/s12955-018-0850-x. 29471823 PMC5822647

[12] D. Coutts‐Bain , L. Sharpe , P. Pradhan , H. Russell , L. C. Heathcote , and D. Costa , “Are Fear of Cancer Recurrence and Fear of Progression Equivalent Constructs?,” Psycho‐Oncology 31, no. 8 (2022): 1381–1389, 10.1002/pon.5944. 35470502 PMC9545421

[13] M. Luchetti , “Epistemic Circularity and Measurement Validity in Quantitative Psychology: Insights From Fechner’s Psychophysics,” Frontiers in Psychology 15 (May 2024): 1354392: PubMed PMID: 38840738; PubMed Central PMCID: PMC11151747, 10.3389/fpsyg.2024.1354392. 38840738 PMC11151747

[14] D. L. Streiner and J. Kottner , “Recommendations for Reporting the Results of Studies of Instrument and Scale Development and Testing,” Journal of Advanced Nursing 70, no. 9 (2014): 1970–1979, 10.1111/jan.12402. 24684713

[15] S. Simard and J. Savard , “Screening and Comorbidity of Clinical Levels of Fear of Cancer Recurrence,” Journal of Cancer Survivorship 1;9, no. 3 (2015): 481–491: September, 10.1007/s11764-015-0424-4. 25603948

[16] C. Maheu , W. L. Tock , P. Fisher , et al. “Systematic Review of Fear of Cancer Recurrence Patient‐Reported Outcome Measures: Evaluating Methodological Quality and Measurement Properties Using the COSMIN Checklist,” Healthcare 13, no. 17 (September 2025): 2165, 10.3390/healthcare13172165. 40941516 PMC12427728

[17] I. R. Podina , D. Todea , and L. A. Fodor , “Fear of Cancer Recurrence and Mental Health: A Comprehensive Meta‐Analysis,” Psycho‐Oncology (2023) [Internet]. [cited 2023 Aug 23], https://onlinelibrary.wiley.com/doi/abs/10.1002/pon.6205. 10.1002/pon.6205 37596855

[18] L. Giguère , B. Mutsaers , C. Harris , et al. “The Ottawa Clinical Fear of Recurrence Instruments: A Screener, Self‐Report, and Clinical Interview,” Psycho‐Oncology 33, no. 6 (2024): e6364, 10.1002/pon.6364. 38824493

[19] B. G. Tabachnick and L. S. Fidell , Using Multivariate Statistics, 5th ed. (Pearson, 2001): Needham Heights.

[20] J. V. Crist and E. A. Grunfeld , “Factors Reported to Influence Fear of Recurrence in Cancer Patients: A Systematic Review,” Psycho‐Oncology 22, no. 5 (May 2013): 978–986, 10.1002/pon.3114. 22674873

[21] M. H. Frost , B. B. Reeve , A. M. Liepa , J. W. Stauffer , and R. D. Hays , “Mayo/FDA Patient‐Reported Outcomes Consensus Meeting Group; What Is Sufficient Evidence for the Reliability and Validity of Patient‐Reported Outcome Measures?,” Value in Health 10, no. 2 (December 2007): 94–105, 10.1111/j.1524-4733.2007.00272.x. 17995479

[22] T. K. Koo and M. Y. Li , “A Guideline of Selecting and Reporting Intraclass Correlation Coefficients for Reliability Research,” Journal of Chiropractic Medicine 15, no. 2 (June 2016): 155–163, 10.1016/j.jcm.2016.02.012. 27330520 PMC4913118

[23] R. L. Gorsuch , Factor Analysis: Classic Edition (Routledge, 2015).

[24] M. Rothmund , M. J. Pilz , N. Egeter , et al., “Patient‐Reported Outcome Measures for Emotional Functioning in Cancer Patients: Content Comparison of the EORTC CAT Core, FACT‐G, HADS, SF‐36, PRO‐CTCAE, and PROMIS Instruments,” Psycho‐Oncology: [Internet]. [cited 2023 Mar 13] (2023), https://onlinelibrary.wiley.com/doi/abs/10.1002/pon.6109. 10.1002/pon.6109 36707461

[25] Boateng G. O. , Neilands T. B. , Frongillo E. A. , Melgar‐Quiñonez H. R. , Young S. L. “Best Practices for Developing and Validating Scales for Health, Social, and Behavioral Research: A Primer.” Frontiers in Public Health [Internet]. 2018 [cited 2022 Apr 19];6, https://www.frontiersin.org/article/10.3389/fpubh.2018.00149. 10.3389/fpubh.2018.00149 PMC6004510 29942800

[26] J. A. E. Custers , S. W. van den Berg , H. W. M. van Laarhoven , E. M. A. Bleiker , M. F. M. Gielissen , and J. B. Prins , “The Cancer Worry Scale: Detecting Fear of Recurrence in Breast Cancer Survivors,” Cancer Nursing 37, no. 1 (2014): E44–E50, 10.1097/ncc.0b013e3182813a17. 23448956

[27] PROMIS . “Anxiety: A Brief Guide to the PROMIS© Anxiety Instruments,”2019, : [Internet], https://www.healthmeasures.net/index.php?option=com_instruments&task=Search.pagination&Itemid=992.

[28] P. A. Pilkonis , S. W. Choi , S. P. Reise , A. M. Stover , W. T. Riley , and D. Cella , “Item Banks for Measuring Emotional Distress From the Patient‐Reported Outcomes Measurement Information System (PROMIS): Depression, Anxiety, and Anger,” Assessment 18, no. 3 (2011): 263–283, 10.1177/1073191111411667. 21697139 PMC3153635

[29] P. A. Pilkonis , L. Yu , N. E. Dodds , K. L. Johnston , C. C. Maihoefer , and S. M. Lawrence , “Validation of the Depression Item Bank From the Patient‐Reported Outcomes Measurement Information System (PROMIS) in a Three‐Month Observational Study,” Journal of Psychiatric Research 56 (2014): 112–119, 10.1016/j.jpsychires.2014.05.010. 24931848 PMC4096965

[30] L. C. Heathcote , S. N. Webster , N. Loecher , et al., “The Bodily Threat Monitoring Scale: Development and Preliminary Validation in Adult and Childhood Cancer Survivors,” Psycho‐Oncology (2023): [Internet]. [cited 2023 Nov 9];n/a(n/a)., https://onlinelibrary.wiley.com/doi/abs/10.1002/pon.6236. 10.1002/pon.6236 PMC11311963 37916988

[31] PROMIS . Depression: A Brief Guide to the PROMIS Depression Instruments, 2019): [Internet], https://www.healthmeasures.net/index.php?option=com_instruments&task=Search.pagination&Itemid=992.

[32] PROMIS . “Life Satisfaction: A Brief Guide to the PROMIS© Life Satisfaction Instruments,”2018, : [Internet], https://www.healthmeasures.net/index.php?option=com_instruments&task=Search.pagination&Itemid=992.

[33] A. S. Gaur and S. S. Gaur , Statistical Methods for Practice and Research: A Guide to Data Analysis Using Spss (Thousand Oaks, 2006), 169.

[34] J. Pallant , SPSS Survival Manual: A Step by Step Guide to Data Analysis Using SPSS, 4th ed. (Open University Press, 2010).

[35] APA Dictionary of Psychology [Internet]. Dictionary.Apa.Org , https://dictionary.apa.org/item‐response‐theory.

[36] T. H. Nguyen , H. R. Han , M. T. Kim , and K. S. Chan , “An Introduction to Item Response Theory for Patient‐Reported Outcome Measurement,” Patient 7, no. 1 (2014): 23–35, 10.1007/s40271-013-0041-0. 24403095 PMC4520411

[37] M. O. Edelen and B. B. Reeve , “Applying Item Response Theory (IRT) Modeling to Questionnaire Development, Evaluation, and Refinement,” Quality of Life Research 16, no. 1 (August 2007): 5–18, 10.1007/s11136-007-9198-0. 17375372

[38] A. Walsh , R. Cao , D. Wong , et al. “Using Item Response Theory (IRT) to Improve the Efficiency of the Simple Clinical Colitis Activity Index (SCCAI) for Patients With Ulcerative Colitis,” BMC Gastroenterology 21, no. 1 (March 2021): 132, 10.1186/s12876-021-01621-y. 33752610 PMC7983213

[39] M. Wiberg , “Can a Multidimensional Test Be Evaluated With Unidimensional Item Response Theory?,” Educational Research and Evaluation 18, no. 4 (May 2012): 307–320, 10.1080/13803611.2012.670416.

[40] M. Ul Hassan and F. Miller , “Discrimination With Unidimensional and Multidimensional Item Response Theory Models for Educational Data,” Communications in Statistics—Simulation and Computation 51, no. 6 (June 2022): 2992–3012, 10.1080/03610918.2019.1705344.

[41] A. F. Hayes and J. J. Coutts , “Use Omega Rather Than Cronbach’s Alpha for Estimating Reliability. But,” Communication Methods and Measures 14, no. 1 (January 2020): 1–24, 10.1080/19312458.2020.1718629.

[42] M. Tavakol and R. Dennick , “Making Sense of Cronbach’s Alpha,” International Journal of Medical Education 2 (June 2011): 53–55, 10.5116/ijme.4dfb.8dfd. 28029643 PMC4205511

[43] D. McNeish , “Thanks Coefficient Alpha, We’Ll Take It From Here,” Psychological Methods 23, no. 3 (2018): 412–433, 10.1037/met0000144. 28557467

[44] Y. Sun , T. T. Luk , M. P. Wang , et al. “The Reliability and Validity of the Chinese Short Warwick‐Edinburgh Mental Well‐Being Scale in the General Population of Hong Kong,” Quality of Life Research 28, no. 10 (October 2019): 2813–2820, 10.1007/s11136-019-02218-5. 31144205

[45] L. Koch , L. Jansen , H. Brenner , and V. Arndt , “Fear of Recurrence and Disease Progression in Long‐Term (≥ 5 Years) Cancer Survivors—A Systematic Review of Quantitative Studies,” Psycho‐Oncology 22, no. 1 (2013): 1–11, 10.1002/pon.3022. 22232030

