# Supplementary material for: Introducing the Ottawa Clinical Fear of Recurrence—Self‐Report; A New Assessment Tool for Clinical Fear of Cancer Recurrence
Source: Psychooncology. 2026 Jul 7;35(7):e70537. doi: 10.1002/pon.70537 (PMC13340200; doi:10.1002/pon.70537)
Supplement: Supplementary file 1 — Supporting Information S1 [file PON-35-e70537-s001.docx]

**Appendix A**

**Item Characteristic Curves (ICCs)**

| **Item** | **IRT** |
| --- | --- |
| 1. I have been distressed when thinking about the possibility of having a cancer recurrence | 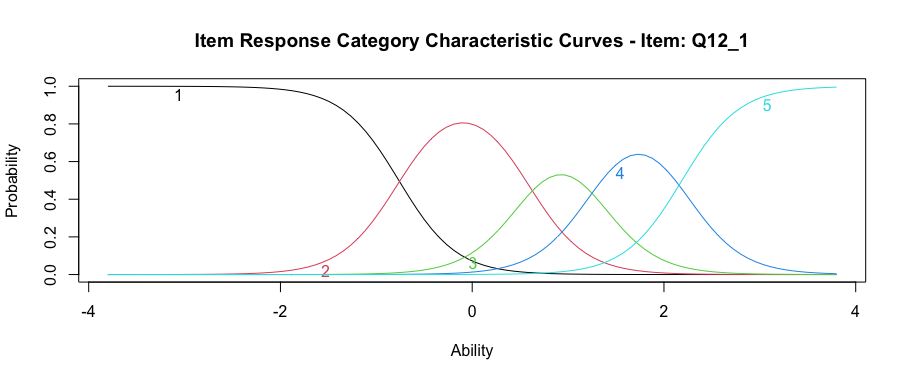 |
| 1. I have images (mental pictures) of myself being ill due to cancer coming back | 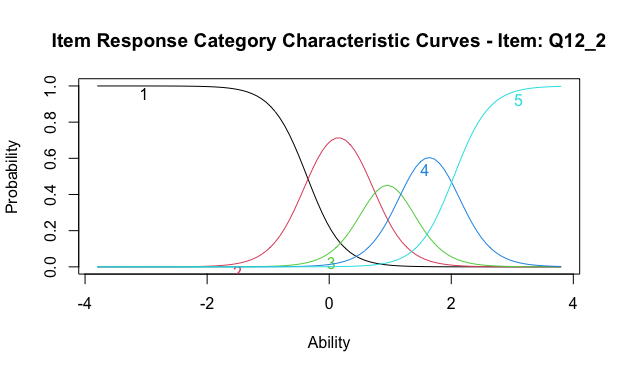 |
| 1. I have concerns about the cancer coming back | 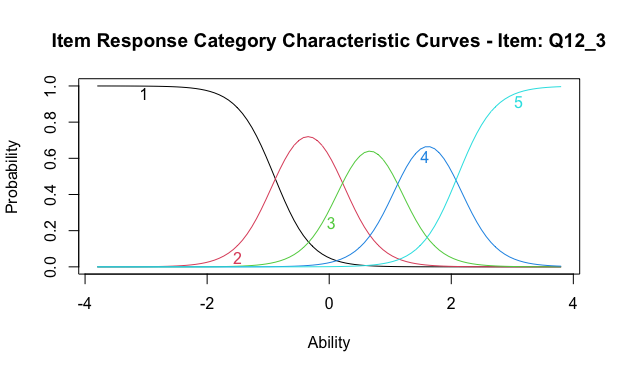 |
| 6. I worry about the possibility of cancer recurrence | 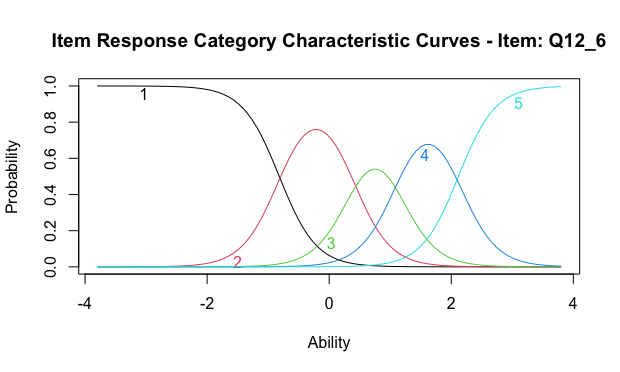 |
| 7. I worry about the possibility of my cancer coming back | 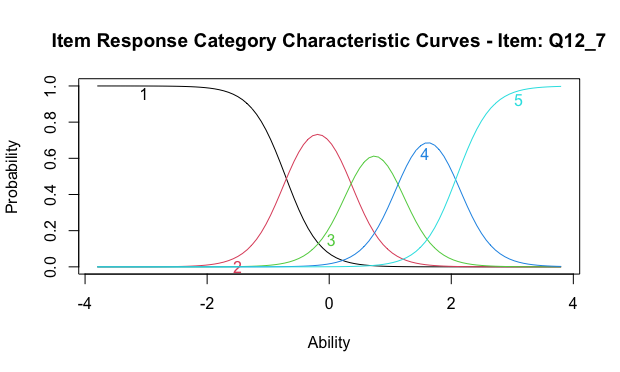 |
| 8. I am afraid of a cancer recurrence | 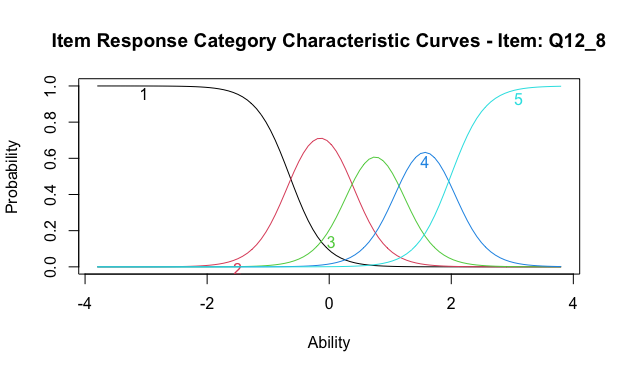 |
| 9. I am anxious about the possibility of cancer recurrence | 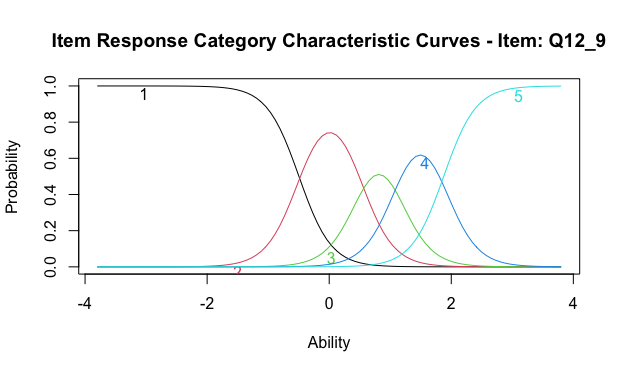 |
| 13. I spend a lot of time examining myself to see if I have any physical signs of cancer | 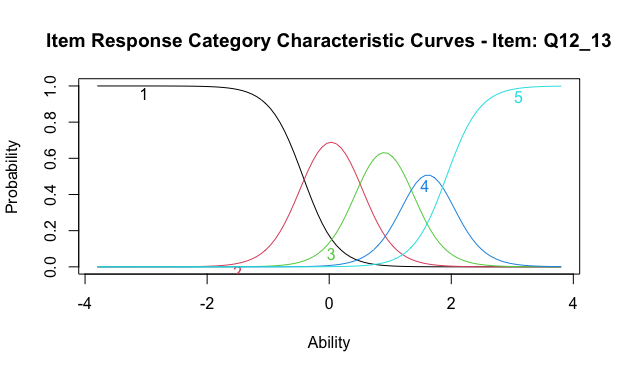 |
| 14. I check my body for signs that my cancer has come back | 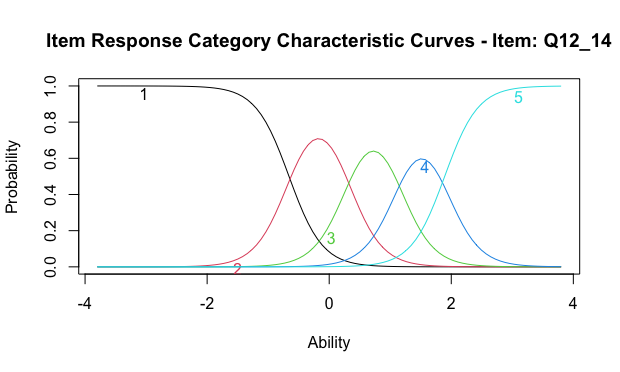 |
| 15. I spend a lot of time seeking out information about signs of cancer recurrence | 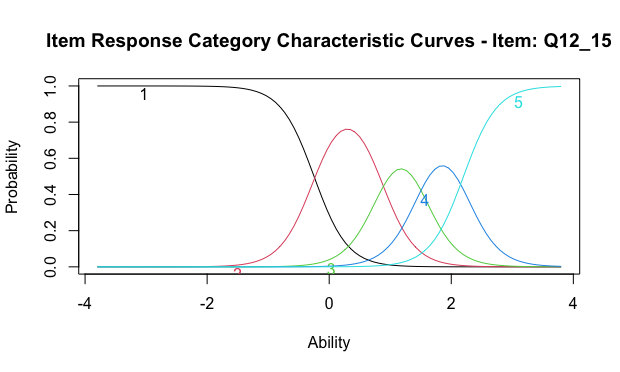 |
| 18. I am unable to work because of my concerns about cancer coming back | 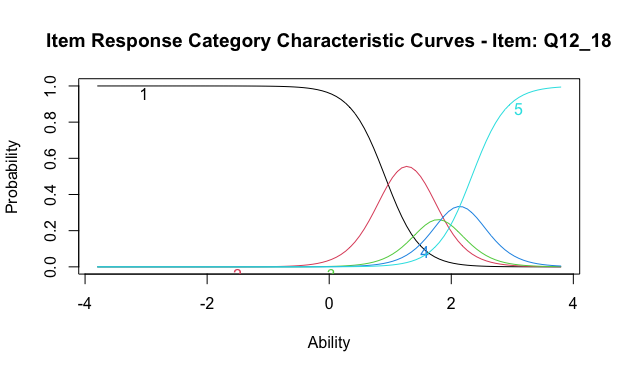 |
| 19. I have difficulty concentrating on what I am doing because of concerns about cancer recurrence | 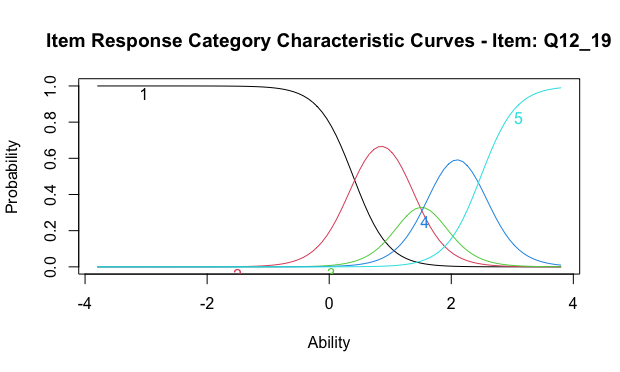 |
| 20. My thoughts or fears about the possibility of cancer recurrence disrupt my work or everyday activities | 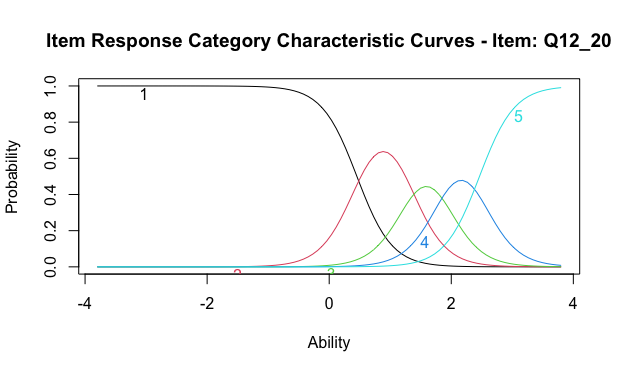 |
| 21. I have trouble sleeping because I cannot get thoughts about cancer out of my mind | 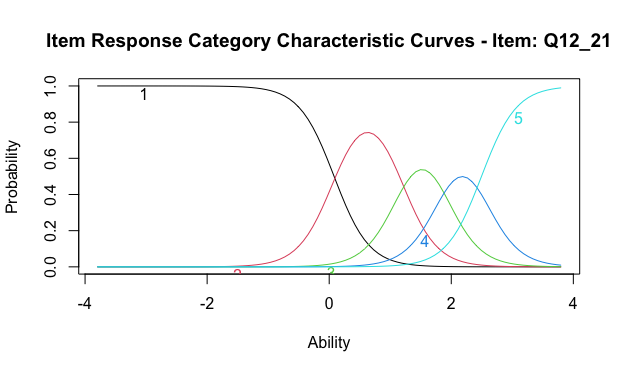 |
| 22. Fear of cancer recurrence interferes with my daily life (e.g., at work, my relationships) | 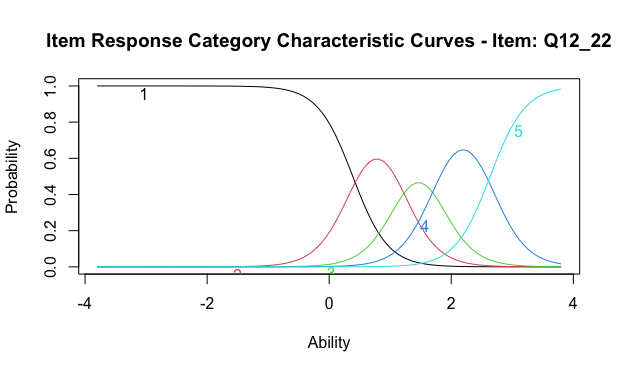 |
| 1. My worries about cancer coming back disrupt my relationships with family, friends, and others who are close to me | 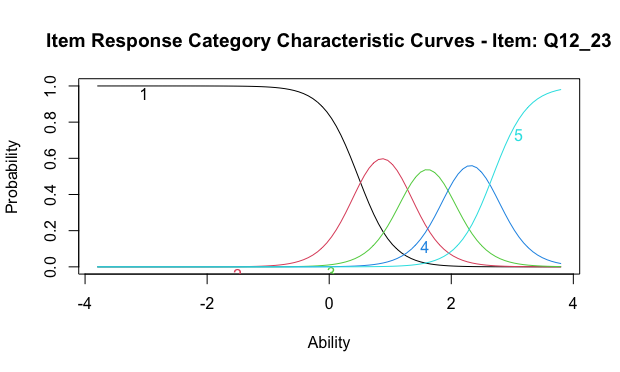 |

**Appendix B**

**Final OCFR-SR scale**

| ***During the past 3 months...*** | **Never** | **Rarely**  (less than once per week) | **Sometimes** (once per week) | **Frequently**  (more than one day per week) | **Almost always** (most days per week) |
| --- | --- | --- | --- | --- | --- |
| 1. I have been distressed when thinking about the possibility of having a cancer recurrence | 0 | 1 | 2 | 3 | 4 |
| 1. I have images (mental pictures) of myself being ill due to cancer coming back | 0 | 1 | 2 | 3 | 4 |
| 1. I worry about the possibility of cancer recurrence | 0 | 1 | 2 | 3 | 4 |
| 1. I spend a lot of time examining myself to see if I have any physical signs of cancer | 0 | 1 | 2 | 3 | 4 |
| 1. I check my body for signs that my cancer has come back | 0 | 1 | 2 | 3 | 4 |
| 1. I have difficulty concentrating on what I am doing because of concerns about cancer recurrence | 0 | 1 | 2 | 3 | 4 |
| 1. Fear of cancer recurrence interferes with my daily life (e.g., at work, my relationships) | 0 | 1 | 2 | 3 | 4 |
